# The Relation between Skin Diseases and the General Health

**Published:** 1892-08

**Authors:** M. B. Hutchins

**Affiliations:** Lecturer on Diseases of the Skin, Atlanta Medical College, Atlanta, Ga.


### THE RELATION BETWEEN SKIN DISEASES AND THE GENERAL HEALTH.*

BY

M. B. HUTCHINS, M. D.,

Lecturer on Diseases of the Skin, Atlanta Medical College,

ATLANTA, GA.

Naturally we seek the causes of skin diseases elsewhere as soon as we become convinced that the "blood" will not suffice. Animal and vegetable parasitic diseases, syphilis, the exanthemata, "new growths" of all kinds, simple or malignant, and diseases which are pretty definitely known to be due to certain micro-organisms are excluded so far as possible from consideration, and we limit our attention to purely eruptive skin disease. A study of the department of "etiology" in works and articles upon skin disease fails to reveal the origin of the "blood disease" theory. We have had the school of "local origin," probably the result of the elder Hebra's teaching and the French (Bazin and Hardy being perhaps the originators), in which various skin eruptions were attributed to the "dartrous diathesis." The soundest doctrine is that which shows a willingness to admit both internal and external factors as causes in many cases, with the reservation that many originate with only one of these factors present ; and the large majority with absolutely no evidence to prove either an internal or external cause which could be identified. My own belief and experience is that we may get the average of a number of persons with or without skin diseases or eruptions, and the percentage of good general health will be as large in one class as the other. Duhring, under "Etiology," says :

"A large proportion of cutaneous diseases are intimately associated with derangement of the internal economy, and there-

*Abstract of a paper read before State Medical Association of Georgia at Columbus, May 20, 1892.

fore strictly speaking symptomatic diseases." I do not think the rest of his work shows that he meant by this that a certain skin disease is always the symptom of a certain internal condition. He says, further, ill health, arising from a variety of causes, plays a conspicuous part both in the causation and continuation of cutaneous affections." From the younger Hebra we get almost an opposite view, in his discussion of the etiology of eczema. He says: "Some attribute all to constitutional causes, others to wholly local influences, but a careful analysis will show that they are due to both. But," he says, "we cannot call eczema a local manifestation of a constitutional disease, as is the case with syphilis." Bulkley says, of eczema: "It is eminently a disease of debility; this may be of three kinds—assimilative, nutritive or nervous, or, as more commonly spoken of, gouty, strumous or neurotic." He thinks that the majority show the gouty. His remarks upon general etiology are worth reproducing.

"Failure in the action of the kidneys, bowels, lungs and liver deranges the balance of the system, and may result in disease of any organ which has work thrown upon it which it cannot perform."

Robinson says: "We do not believe that at any time such conditions as scrofula, anæmia, chlorosis, etc., produce eczema, except in an indirect way by increasing the irritability of the elements of the skin, or lessening their power to withstand direct irritation either from within or without, and we are obliged to hold eczema of local origin." What Fox says of eczema applies to other skin eruptions, viz.: "Its association with various maladies may suggest a relationship." Here are a few of the "predisposing" causes of eczema as enumerated by Veiel in Ziemssen's Handbook of Skin Diseases, viz.: Chlorosis, rachitis, scrofula, gout, albuminuria, diabetes, gastric and intestinal catarrhs, dysmenorrhœa, uterine affections, lactation, pregnancy—and if he has omitted any we must believe it was unintentional. The whole matter could have been as fully expressed by saying, "Any condition of health which tends to lower the vital forces and reduce the powers of resistance of the skin may act as a predisposing element." Eichhorst says, in Wood's Library, in speaking of causes of eczema, "There is danger of mistaking an accidental complication for an etiological factor." Elliot speaks of the "indefinite and deplorably termed condition 'impurity of the blood'" in his article on the treatment of eczema.—*Med. and Surg. Jour.*, 1890.

Piffard believes that fully one-third of all skin diseases originate from a *materies morbi* generated in the body from digestive, assimilative or excretory function. He further thinks that the most peccant matters in eczema are uric and oxalic acids. Taylor says that "the attempt to prove that a number of distinct morbid conditions or diatheses is at the root of eczema and allied skin diseases has resulted in failure." Robinson says that "dentition is no more the cause of skin disease than of other troubles innocently charged to it."

From my own notes of about three hundred cases I eliminate about sixty as not properly coming under consideration, belonging to one or other of the classes mentioned at the beginning of this paper. Of the remainder, I find about eighty-seven had some general disorder, and about a dozen only chronic constipation. The age ranged from two months to eighty-five years. The skin diseases among the number were those most commonly met with in practice. Chronic urticaria alone (three cases) seemed always accompanied by the rheumatic diathesis. The most common deviation from health was some form of dyspepsia in adults, struma and bowel trouble in children, occasional excess of uric acid in the urine of either. Children whose nutrition was low, owing to artificial feeding, were generally troublesome cases to treat. The urine of patients was not regularly examined unless it seemed necessary, because I tested and examined the urine of every patient who came into the New York Skin and Cancer Hospital until I got tired of negative results—save occasional uric acid, oxalate of lime or phosphates in excess—and became convinced that nothing abnormal would be found in the urine of the average skin patient. I have seen a patient with eczema get a severe relapse after taking a few bottles of beer, and I have had cases of women which were somewhat aggravated at the periods. But it would be a useless waste of time to read the details of my cases. It is sufficient to say that there were at least one-third of the true eruptive cases in which it was impossible to ascertain any deviation from health, and it is also true that the majority of those cases in which there was a deviation from health showed no positive evidence of a direct connection between the skin trouble and the internal disease.

The diagnosis of a skin disease is as important as the diagnosis of any other disease, for the treatment of different diseases differs markedly. The prognosis depends upon the correct treatment, the faithful following of directions by the patient and our ability to restore the vital functions to a conditon rendering it possible for the skin to receive its proper nutriment or its proper nervous influence. You must select your cases in giving arsenic. You will find some other diseases besides syphilis which may sometimes be favorably influenced by iodide of potassium, perhaps through its diuretic action. There is a common belief that it is dangerous to cure certain skin diseases lest you "drive it back in the blood," as a patient told me. This is related to the idea that *fistula in ano* should not be cured lest the patient develop consumption; a belief which no longer universally exists.

Duhring says it should always be the aim of the physician to cure a skin disease as rapidly and effectively as possible.

Crocker says: "The popular idea that it is dangerous to cure eruptions quickly, or, as the laity put it, 'drive the rash in," is as erroneous as the notion that nearly all skin diseases are due to 'impurities in the blood.'" Taylor mentions the absurdity of the idea of "danger in curing up."

Simon says, "Hebra was no doubt right when he said there was no specific constitutional treatment for skin diseases." Crocker says: "Probably of all conditions requiring attention, dyspepsia and other disorders of the alimentary canal are the most important." Elliot says: "There are no specifics for eczema, no drugs which influence the process directly, and in virtue of their peculiar properties and action, but the benefit which is obtained from the use of any medical agent internally is through its power or capability of removing or improving those functional or other disturbances of health which brought about a predisposing and favorable condition for the development of the disease. The whole matter of treatment can be expressed in a few words: Treat the general condition just as if there were no skin disease present, and the skin disease with appropriate local application.

To sum up now: I have written mostly of eczema because it is the best known and most common skin disease—comprising about one third of all. You find nearly every type of skin disease in one or other form of eczema; and what applies, in general, to eczema applies to all the others.

Two things are evident after reading the opinions of the various writers upon skin diseases: First, we can be positive that skin diseases are not all "blood diseases," or the idea would have been universally accepted. In the second place, the unanimity of authorities in attributing various of these diseases indefinitely to some "nervous influence," and their disagreement in many details, show that there is yet much to be learned concerning the actual etiology of skin diseases. The "germ theory" has received scant attention in this paper, because the discoveries of skin disease producing "germs" are yet in such a decided condition of infancy that a discussion thereupon would lead us further into the dark than the necessities of the case demand.

